# Dietary Diversity among Children Aged 6–23 Months in Aleta Wondo District, Southern Ethiopia

**DOI:** 10.1155/2019/2869424

**Published:** 2019-11-13

**Authors:** Karisa Dafursa, Samson Gebremedhin

**Affiliations:** ^1^Sidama Zone Health Department, Hawassa University, P.O. Box 51, Hawassa, Ethiopia; ^2^School of Public Health, Addis Ababa University, P.O. Box 05, Addis Ababa, Ethiopia

## Abstract

**Background:**

Dietary diversity (DD) is among the core infant and young child feeding (IYCF) indicators. However, in many developing countries, meeting the minimum standards of DD is challenging and information concerning its determinants is limited.

**Objective:**

To assess the level and predictors of DD among children aged 6–23 months in rural communities of Aleta Wondo district, Sidama zone, Southern Ethiopia.

**Method:**

A community-based cross-sectional study was conducted in rural Aleta Wondo in February 2016. Multistage sampling was employed to recruit 502 children aged 6–23 months. DD was assessed by asking the mother whether the index child had received food from the standard seven food groups in the previous day, without setting minimum intake restrictions. Ultimately, the dietary diversity score (DDS) was rated on a 7-point scale, and it was modeled using linear regression analysis. The outputs are presented using adjusted regression coefficients (*β*).

**Results:**

Only 12.0% (95% confidence interval: 9.0–15.0%) of the children met the minimum recommended DD, receiving from four or more from seven food groups. The analysis identified eleven significant predictors of DDS. As the maternal knowledge of IYCF increases by a unit, DDS raised by 0.21 units (*p* = 0.004). Unit increment in the husband's involvement in the IYCF score was linked with 0.32 units improvement in DDS (*p* = 0.016). One unit change in the ordinal category of household food insecurity was associated with 0.13 reduction in DDS (*p* = 0.001). Similarly, household wealth index (*β* = 0.54, *p* = 0.041), father's literacy (*β* = 0.48, *p* = 0.002), ownership of home garden (*β* = 0.38, *p* = 0.01), mother's participation in cooking demonstrations (*β* = 0.19, *p* = 0.036), and child age in months (*β* = 0.04, *p* = 0.001) were all positively associated with DDS. Furthermore, receiving IYCF information via mass media (*β* = 0.04, *p* = 0.001) and during antenatal (*β* = 0.91, *p* = 0.022) and postnatal checkups (*β* = 0.21, *p* = 0.043) were positive predictors of DDS.

**Conclusions:**

Promoting the socioeconomic status of the community, strengthening of home gardening, involving husbands in IYCF, and enhancing maternal knowledge of IYCF may advance DD.

## 1. Background

Child undernutrition remains a critical public health challenge in the world [1, 2]. According to the 2016 estimate of the World Health Organization (WHO), globally 22.9% (155 million) children under five years are stunted and 7.7% (55 million) are wasted. Sub-Saharan Africa (SSA)—with 40% prevalence of stunting and 7.4% prevalence of wasting—has the highest burden of child malnutrition in the world [1]. Between 2000 and 2016, SSA experienced a slower progress in reducing stunting, and the actual number of stunted children has risen [1, 3]. In Ethiopia, according to the Demographic and Health Survey (DHS) 2016, 38, 24, and 10% of children under the age of five years are stunted, underweight, or wasted, respectively [4].

Amongst the leading health risk factors, undernutrition remains the major cause of mortality and disability-adjusted life years (DALYs) loss in children [5]. Every year undernutrition contributes to the death of 3 million children and affects the physical and mental development of millions of others [6]. It is estimated that about one-third of the global death of children under the age of 5 years is indirectly attributable to undernutrition [7]. Furthermore, 11% of the global DALY loss can be averted by preventing macronutrient and common micronutrient deficiencies [5].

The complementary feeding period, which typically extends between 6 and 23 months of age, is characterized by a gradual transition from breast milk to family food. It is a vulnerable period with peak incidence of growth faltering, as well as the occurrence of micronutrient deficiencies and infectious diseases [7, 8]. Complementary foods often have inadequate quantity and nutrient density, prepared in an unhygienic way, and are initiated too early or too late [7]. In the developing world, age-appropriate, nutritious, and hygienic child feeding coupled with optimal breastfeeding has the potential to prevent one-fifth of all childhood deaths [7]. The 2008 Lancet series estimated that about 6% of childhood deaths can be averted by assuring optimal complementary feeding [9].

Dietary diversity (DD) refers to the number of various food groups consumed by an individual or members of a household over a reference period, irrespective of the frequency of consumption, and with or without imposing a minimum intake restriction [10]. The reference period may range from 1 to 15 days, and the numbers of food groups of interest may vary from 7 to 14 [10, 11]. Dietary diversity is a simple tool frequently employed as a proxy measure of dietary quality, micronutrient adequacy, and food access. Reasonable number of studies has validated the utility of DD for predicting micronutrient intake and the nutritional status of children [12–14]. In 2008, the WHO proposed DD as one of the eight core indicators for assessing infant and young child feeding (IYCF) practices in population-based surveys [15]. In IYCF context, optimal DD is defined as consuming from 4 or more groups in the previous day, out of the standard 7 food groups, without imposing a minimum intake restriction.

Meeting the minimum standards of DD for infants and young children remains a major challenge in many developing countries including Ethiopia. In Ethiopia, according to the recent DHS—2016, only 14% of children aged 6–23 months met the minimum standards of DD despite nearly half (45%) of them having optimal meal frequency [4]. Furthermore, the three major DHS surveys conducted in Ethiopia since 2000 witnessed no significant progress in the dietary diversity score (DDS) of infants and young children over the last 15 years [4]. Small-scale surveys from various parts of the country also came to the same conclusion [16–18]. However, limited information exists on what predicts the DD of infants and young children.

Therefore, this study aimed at assessing the level of DD and associated factors among children aged 6–23 months, in rural Aleta Wondo district, Sidama Zone, Southern Ethiopia.

## 2. Materials and Methods

### 2.1. Study Area

The study was conducted among mothers of children aged 6–23 months living in the Aleta district, Sidama zone. The capital of the district Aleta Wondo town is located 330 km south of Addis Ababa. According to a 2015 estimate, the district has a population of 205,000, of whom 89% dwell in rural areas and 12,729 (6.2%) were children under the age of two years. The vast majority of the inhabitants are Sidama in ethnicity (92%) and are affiliated to Protestant Christianity (73%).

Administratively, the district is organized into 2 urban and 27 rural villages—the smallest administrative unit in Ethiopia comprising approximately 1,000 households. The Aleta Wondo district has an area of 210 km^2^, and more than 70% of the land is considered arable. The inhabitants are mainly reliant on subsistence agriculture, and the major crops grown are maize, root crops especially *Ensete* (false banana), haricot bean, and cash crops such as coffee and *Khat*. Regarding access to health services, the district has 7 health centers and 27 health posts.

### 2.2. Study Design

A community-based cross-sectional study with both descriptive and analytic elements was conducted in February 2016.

### 2.3. Study Participants

All children aged 6–23 months who were permanent residents of the 27 rural villages of Aleta Wondo district were considered as the source population of the study, while children in 8 rural randomly selected villages were considered as the study population.

### 2.4. Sample Size

The sample size for determining the percentage of children aged 6–23 months who met the minimum DD was estimated as 509 using single population proportion formula [19]. The computation was made assuming 10.6% expected proportion [16], 95% confidence level, 4% margin of error, design effect of 2, and 10% compensation for possible nonresponse.

On the contrary, a sample size of 109 was considered optimal for identifying determinants of DDS. The computation was made using G^∗^power software [20] assuming the data analysis would be made via the multivariable linear regression model based on 22 predictors. Other specifications made during the computation were 95% confidence level, 90% power, 0.3 (medium) effect size, and 10% contingency for possible nonresponse. Accordingly, the largest sample size (509) was taken as the ultimate sample size of the study.

### 2.5. Sampling Techniques

The study employed the multistage cluster sampling technique for identifying the study subjects. Initially, the 27 rural villages were stratified into two agroecological zones: highland and midland based on their altitude above sea level (ASL). villages located 1,500 to 2,300 and above 2,300 meters ASL were considered as having midland and highland agroecology, respectively. From the available 23 midland and 4 highland villages, 6 and 2 villages, respectively, were selected using a lottery method. The total sample size (*n* = 509) was proportionally distributed to the 8 selected villages in consideration of their population size. Then, in each villages, exhaustive listing of the eligible children was made by engaging the local health development army (HDA) members, and the list was used as the sampling frame of the study. Ultimately, the required number of children was selected using a systematic sampling technique.

### 2.6. Data Collection Tools and Procedures

The data were gathered by eight trained data collectors and two field supervisors using a pretested and structured questionnaire. The tool was developed in English, translated into the local Sidama language, and back translated to English to check its consistency. Sociodemographic, economic, and IYCF-related questions were directly adopted from the standard DHS questionnaire [21].

Dietary diversity was assessed by asking the mother whether the child had received food from the standard seven food groups in the preceding day, without setting minimum intake restrictions [15]. The seven food groups were grains, roots, and tubers; legumes and nuts; dairy products excluding breast milk; flesh foods (meat, fish, poultry, or organ meats); eggs; vitamin A-rich fruits and vegetables; and other fruits and vegetables. A dietary diversity score (DDS), which ranges from 0 to 7, was computed. Children who received at least 4 of the 7 food groups in the reference period were considered to have met the minimum DD [15].

Meal frequency was measured as a proxy indicator of calorie intake in accordance with the recommendation of the WHO [15]. Children aged 6–8 months who received at least two meals and children aged 9–23 who received 3 or more meals in the preceding day were assumed to have met the minimum meal frequency standard [15]. Children who had satisfied both the minimum standards for DD and meal frequency were considered to have an acceptable diet [15].

Continued breastfeeding rate at one and two years was estimated based on proportion of children aged 12–15 and 20–23 months, respectively, who received breast milk in the preceding day. Timely introduction of complementary foods was determined based on proportion of children aged 6–8 months of age who received solid, semisolid, or soft foods in the previous day. Similarly, age-appropriate breastfeeding was estimated based on the percentage of children who received breast milk, as well as solid, semisolid, or soft foods, during the previous day [15].

Household food security was measured using Household Food Insecurity and Access Scale (HFIAS) based on the frequency of occurrence of nine food insecurity-related events in the preceding 4 weeks. The scale classifies the extent of food insecurity into four ordinal categories: food secure and mild, moderate, and severe insecurity [22].

Mothers' knowledge of IYCF was assessed based on their response to ten questions developed by the investigators. The questions were focused on issues including optimal duration of exclusive and total breastfeeding; appropriate time for introducing complementary food; dietary diversity; and opinions on feeding infants and young children with animal source foods. Right responses were coded as 1, and all other responses were coded as 0. Ultimately, it was scored on a 10-point composite scale. The questions used for assessing the mother's knowledge on IYCF are provided as a supporting file with this article (Supporting [Supplementary-material supplementary-material-1]).

Husband involvement in IYCF was measured based on the response of the mothers to seven questions pertaining their husbands' support in child feeding. These include practice of the husband in terms of discussing child feeding issues at home, availing money to buy special foods (animal source foods) for the baby, bringing special foods home, supporting the mother while preparing meals for the baby, feeding the child himself, supporting the mother in domestic chores while she engages in food preparation or child feeding, and following and encouraging her for proper child feeding. Positive practices were coded as 1, and the rest were coded as 0. Ultimately, it was scored on an 8-point composite scale.

### 2.7. Study Variables

**Figure 1:**
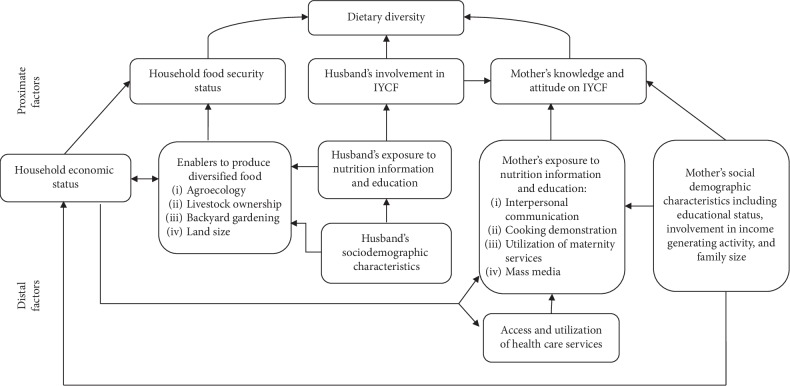
Conceptual framework of the study.

As depicted in Figure 1, the study considered various predictors of DDS. These include sociodemographic characteristics of the mother (age, educational status, marital status, and involvement in income-generating activities), educational status of the father of the child, socioeconomic status of the household including wealth index, household food insecurity and land size, age and sex profile of the index child, number of children under the age of five years in the household, agroecology of the village, maternal knowledge of IYCF, husband's involvement in IYCF, ownership of livestock and home garden, exposure to nutrition counseling and education through mass media and interpersonal communication with health extension workers (HEWs) and HDA members, participation in cooking demonstrations, and exposure to IYCF information during antenatal (ANC) and postnatal (PNC) visits (Figure 1).

### 2.8. Data Management and Analysis

Data entry was made using EPi Info 7 software and exported to SPSS 20 for analysis. Frequency distribution, measures of central tendency, and dispersion were used to summarize the data. Core and selected optional IYCF indicators were computed as recommended in the WHO guideline [15].

Wealth index was computed as a measure of household wealth using principal component analysis (PCA). Fifteen variables related to ownership of selected household assets, size of agricultural land, quantity of livestock, materials used for housing construction, and ownership of improved water and sanitation facilities were considered. Finally, the generated principal component was divided into 5 equal quintiles (lowest, second, middle, fourth, and highest).

Bivariable and multivariable linear regression analyses were used to model DDS. All explanatory variables that demonstrated a *p* value less than 0.25 in bivariable analysis were considered as candidates for the multivariable models. In order to avoid overadjustment bias and unnecessary adjustment, independent variables were fitted into two different distal and proximate models in accordance with the conceptual framework of the study [23]. The outputs of the analyses are presented via crude and adjusted unstandardized regression coefficients (*β*).

In final multivariate linear regression models, the extent of multicollinearity was measured using variance inflation factor (VIF) and found to be within tolerable range (less than 10). Linearity of the association and normality, homoscedasticity, and independence of the error terms were evaluated using partial plots. The goodness-of-fit of the models was assessed using the *F*-test and adjusted *R*-squared value.

### 2.9. Ethical Consideration

The study was cleared by the Institutional Review Board of College of Medicine and Health Sciences, Hawassa University. Permissions were taken from regional, zonal, and district health offices. Data were collected after taking informed written consent from the mothers. All information gathered was kept confidential. At the end of the survey, mothers who were providing a poorly diversified diet to their children were given nutrition education.

## 3. Results

### 3.1. Sociodemographic Characteristics

Among 509 mothers approached, 502 (98.6%) consented to take part in the study. Of infants and young children enrolled, the male-to-female ratio was 1.12. The mean (±SD) age of the children was 14.7 (±5.5) months, and 69.5% were aged between 12 and 23 months.

The mean (±SD) age of mothers was 26.9 (±5.3) years, and more than half (55.0%) were within the range of 25–34 years. Furthermore, about three-fourths (76.5%) were illiterate, 89.0% were housewives, and nearly all (96.4%) were married. Regarding their partners, 73.7% were illiterate and 82.7% identified farming as their occupation.

The vast majority (98.2%) of the children were sampled from male-headed households. The mean (±SD) family size of the represented households was 5.1 (±1.6), and more than half (58.0%) had 5 or more members. Similarly, 51.4% had two or more children under the age of five years (Table 1).

### 3.2. Household Agricultural Production and Food Security

About four-fifths (80.1%) of the respondents were sampled from the midland agroecology zone, while the remainder were highlanders. Almost all of the households (98.8%) owned a plot of land for agricultural purpose, and the mean (±SD) land size was 1.2 (±0.5) hectares. Domestic production was reported as the major source of food in 77.5% of the households. Commonly produced crops were *Ensete* (false banana) (97.4%), cash crops like coffee or *Khat* (87.5%), and vegetables (60.2%). Furthermore, two-thirds (67.7%) own livestock. Food security assessment based on HFIAS revealed that nearly half (48.4%) of the households were food insecure (Table 2).

### 3.3. Women's Access to Nutrition Information and Education

About 56.8, 35.9, and 35.1% of the mothers received IYCF-related information or education from HEWs, health professionals, and HDA members, in the preceding one month of the study, respectively. Nearly half (46.4%) heard IYCF messages from the mass media in the same reference period. One-fifth (21.7%) reportedly participated in complementary food cooking demonstrations that were organized in the preceding 6 months. The majority were exposed to IYCF messages during antenatal (ANC) (88.6%) and postnatal (78.9%) checkups.

### 3.4. Infant and Young Children Feeding Practices

**Figure 2:**
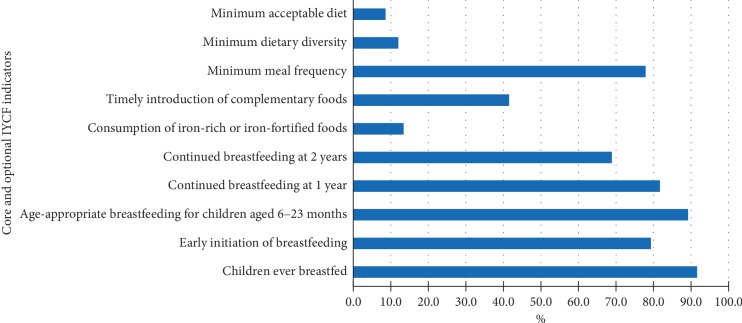
Summary of core and optimal IYCF indicators, Aleta Wondo district, Southern Ethiopia.

About 91.6% of the children were ever breastfed, 79.3% put to the breast within one hour of birth and 79.6% received colostrum. Children under 6–8 months, less than half (41.5%), were fed solid, semisolid, or soft foods during the previous day. A reasonably high proportion of children continued breastfeeding at one (81.7%) and two years (68.9%). Only 13.6% received iron-rich or iron-fortified foods designed for infants and young children. The vast majority (89.2%) of children aged 6–23 months had age-appropriate breastfeeding (Figure 2).

### 3.5. Level of Dietary Diversity and Meal Frequency

The mean (±SD) DDS of the children in the preceding day was 2.5 (±0.9), and only a small proportion (12.0%: 95% CI: 9.0–15.0%) met the minimum requirement for DD. Grains, roots, and tubers (89.0%) and milk and milk products (72.5%) were the most commonly consumed food groups. Conversely, eggs (21.5%), vitamin A-rich fruits and vegetables (21.5%), legumes (17.1%), other fruits and vegetables (11.8%), and flesh foods (3.4%) were less frequently consumed.

More than three-fourths (77.9%) of the children received solid, semisolid, or soft foods, the minimum recommended number of times or more during the previous day. Of the children, less than one-tenth (8.6%) met the minimum acceptable diet—combination of minimum DD and minimum meal frequency.

### 3.6. Factors Associated with Dietary Diversity Score

A total of 22 independent (3 proximate and 18 distal) variables listed earlier were considered as predictors of DDS. The association of each predictor with the response variable was evaluated using bivariable linear regression analyses. Fifteen variables had *p* values less than 0.25 and hence were considered as candidate variables for multivariate analyses. In the multivariate model, eleven demonstrated a significant association with DDS (Table 3).

In the proximate multivariable model, all the three variables showed significant associations with the outcome variable. As the maternal knowledge of IYCF increased by one unit, DDS increases by 0.21 units (*p*=0.004). Unit increment in the husband's involvement in the IYCF score was linked with 0.32 units increment in DDS (*p*=0.016). A one unit shift in the ordinal category of household food insecurity was associated with 0.13 units decline in DDS (*p*=0.001).

In the distal multivariable model, 8 variables showed significant associations with DDS. Unit change in the household wealth index was associated with 0.54 (*p*=0.041) rise in DDS. Among children having literate fathers, the DDS was increased by 0.48 (*p*=0.002) units. Ownership of a backyard garden (*β* = 0.38, *p*=0.01), mothers participation in cooking demonstrations (*β* = 0.19, *p*=0.036), receiving IYCF information from the mass media (*β* = 0.04, *p*=0.001), and during ANC (*β* = 0.91, *p* = 0.022) and PNC (*β* = 0.21, *p*=0.043) were associated with improved DDS. A significant positive association was also observed between child age and DDS (*β* = 0.04, *p*=0.001).

## 4. Discussion

This study demonstrates that only a small proportion of children in the district met the minimum requirement for dietary diversity, and the level was even inferior to the 2015 target set by the Ethiopia National Nutrition Program (NNP) [24]. DDS showed a significant association with numerous factors including household wealth and food security status, husband's literacy and involvement in IYCF, maternal knowledge of IYCF, ownership of a backyard garden, and receiving IYCF information through the mass media, cooking demonstrations, and interpersonal communication while receiving maternity care.

Only 12.0% of the children aged 6–23 months satisfied the minimum DD, and this is comparable with a number of other studies conducted in Ethiopia. The Ethiopian DHS 2016 concluded that only 14% of children received adequately diversified diet, and regional figures ranged from 2.6% in Afar to 43% in Addis Ababa [4]. The figures reported by other local studies in South Wollo (7%) [25], Abiy Adi (10.8%) [26], Gorche (11%) [16], Dangila town (12.6%) [27], Damot Sore (17%) [28], and Kemba (23.3%) [29] were also consistently low. This indicates meeting the minimum required food diversity has remained a challenge throughout the country.

The majority of the children (89%) consumed grains, roots, and tubers, while consumptions of eggs, legumes, and flesh foods were low. A study conducted in largely food insecure areas of the South Wollo zone, Northern Ethiopia, concluded that children frequently receive starchy staples (90%), while consumption of proteinous foods including legumes was low [25]. Many other local studies documented similar consumption patterns [4, 16, 27, 28]. The low consumption of protein-rich foods can be due multifold factors including lack of nutritional awareness and shortage of access due to economic constraints [25].

The study observed that household economic status—as measured by wealth index and food security level—was a significant predictor of DDS. Intuitively, the inferior economic status restricts the availability and variety of food in the household. The Ethiopian DHS 2016 documented that the proportion of children who received the minimum required DD consistently increased from 8% in the lowest to 28% in the richest wealth quintiles [4]. In South Wollo, children from households with moderate and severe food insecurity had 9% and 25% reduced likelihood of getting diversified diet, respectively [25]. Due to the strong association frequently observed between food security and DD, some studies proposed DDS as an index of household food insecurity [10, 30].

Our study suggests that husbands' literacy and involvement in IYCF enhance the variety of food offered to children. A study conducted in the South Wollo zone, Northern Ethiopia, concluded that husbands' direct involvement in IYCF augments DDS by 13.7% [25]. Similarly, an undertaking in a nearby Gorche district reported that husbands' engagement increased DD by 0.2 scores [16]. In patriarchal societies like that of Aleta Wondo, though indoor chores including cooking and child feeding are left for mothers, fathers remain the decision makers on the overall financial and food supply to the household. Accordingly, they play an enabling role to their wives. Furthermore, males with positive deviance from the norm may effectively influence the behavior of their partners.

There is a reasonable body of evidence on the link between maternal knowledge of IYCF and the quality of the diet offered to children. Likewise, our study found that maternal knowledge is positively associated with DDS. Though the exiting literature employed assorted approaches to measure and classify maternal knowledge, local studies conducted in South Wollo [25], Gorche [16], Abiy Adi Northern Ethiopia [26], and Dangila town [27] ended up with similar conclusions. In the South Wollo zone, DDS in children born to knowledgeable mothers was increased by 3% [25]. In Gorche, unit change in the maternal knowledge score was linked with 0.4 rise in DDS [16].

The utilization of maternity services in Ethiopia remains low [4]. Our study found mothers who received IYCF information during ANC and PNC checkups are likely to offer food to their young children from four or more food groups. Parallel findings have been reported by studies conducted in Ethiopia [29, 31, 32] and elsewhere [33]. This may imply that promoting the utilization of maternity services and stronger integration with IYCF helps to improve infant feeding practices. Conversely, the observed association can also be explained by extraneous factors. Mothers who have attended ANC and PNC visits may have better access to services or might be from a well-off family and thus are more likely to provide a diversified diet to their children [33].

In our study, nearly half of the mothers heard IYCF messages from the mass media and those who had such exposure were more likely to offer a diversified diet to their children. Studies conducted in Ethiopia [16, 25, 27], Kenya [34], and Nepal [33] made the same observations. This is not a surprising finding because the mass media is considered to be a trustworthy source of information that can affect behavior. In particular, the finding makes sense considering the fact that IYCF-related radio and TV spots are recently becoming more frequent and popular in Ethiopia. Conversely, as mothers who have access to the media are likely to be from a well-to-do family, the observed association can also be due to the residual confounding effect of household wealth status.

The other factor that turned out to be a significant predictor of dietary diversity was mothers' involvement in complementary food cooking demonstrations. Multiple studies from Ethiopia [16, 25], Peru [35], and India [36] came up with a similar conclusion. The finding is consistent with the understanding that a cooking demonstration specially benefits socioeconomically disadvantaged rural communities through providing practical experiences on how to prepare complementary foods using locally available and cheaper ingredients [25].

The existing national and international IYCF guidelines propose that children aged 6–23 months should daily receive food from 4 or more food groups irrespective of their age [15, 24]. Yet our study observed that the variety of foods given to younger children is even lower and DDS only tends to improve with increasing age. Similar patterns have also been observed in Ethiopia [16, 25–27] and other developing countries [33, 37]. This could be because caregivers may assume that younger infants do not need diversified food or their gut may not be able to digest animal source foods. Consequently, complementary feeding might be initiated with monotonous staples. A study from Northern Ethiopia found flesh foods and eggs are typically introduced in children's diets in the middle of the second year of age [25]. A study in Nepal that compared the pattern of consumption of basic food groups across different age categories concluded that, for most types of food, especially for meat, fruits, and vegetables, the biggest improvement in consumption occurs between 12 and 17 months of age [33].

We found that children from households with home gardens were likely to receive food from four or more food groups. This supports the findings of studies conducted in Ethiopia [16, 25, 27, 31] and other developing countries [38, 39]. Home gardening may promote dietary diversification through enhancing access to fruits and vegetables and sometimes to root and tuber staples. Furthermore, it may indirectly improve child feeding by advancing household food security and women empowerment [39].

The study has some limitations that should be considered. First, the reported DD was assessed based on single day recall; hence, it may not exactly reflect the common dietary practices of the children. It may, however, give a reasonable picture on what is going on at the population level. Furthermore, the figure might have also been over- or underestimated due to recall and social desirability bias. The second limitation is that, due to absence of standardized tools, mother's knowledge of IYCF and husband's involvement in child feeding were assessed using a set of questions that has not been validated. This might have resulted in random misclassification bias. Thirdly, dietary intake of the child can be affected by the appetite and illness status of the child, but such variables were not accounted for in the study. Finally, ownership of backyard garden and livestock were treated as dichotomous variables, and no attempt was made to measure the backyard plot size or the number of livestock. This may result in underestimation of the association. Furthermore, as is the case with many other observational studies where attempts were made to control multiple confounders using regression models, we cannot entirely exclude residual confounding.

## 5. Conclusion

The minimum recommended dietary diversity was only achieved by 12% of the infants and young children. Low household socioeconomic status as well as lack of nutritional knowledge led to limited provision of a variety of food to children. Predictors of DDS include household wealth and food security status, husband's literacy and involvement in IYCF, maternal knowledge of IYCF, ownership of a home garden, and receiving IYCF information through multiple modalities including the mass media and cooking demonstrations [40].

Promoting the socioeconomic status of the community, strengthening of home gardening, engaging husbands in IYCF, and enhancing maternal knowledge of IYCF via multiple modalities including the mass media and organization of cooking demonstrations can serve as effective vehicles for improving dietary diversity of infants and young children.

## Figures and Tables

**Table 1:** Sociodemographic characteristics of children aged between 6 and 23 months in the Aleta Wondo district, Southern Ethiopia.

Variables (*n* = 502)	Frequency	Percentage
Child's age (months)		
6–8	82	16.3
9–11	71	14.2
12–23	349	69.5
Sex of the child		
Male	266	53.0
Female	236	47.0
Age of the mother (years)		
15–24	176	35.1
25–34	276	55.0
35–49	50	10.0
Mother's educational status		
Illiterate	384	76.5
Literate	118	23.5
Mother's occupation		
Housewife	447	89.0
Farmer	21	4.2
Government employee	17	3.4
Merchant	17	3.4
Religion		
Protestant	465	92.6
Catholic	17	3.4
Others	20	4.0
Marital status		
Married	484	96.4
Others	18	3.6
Father's educational status		
Illiterate	370	73.7
Literate	132	16.3
Father's occupation		
Farmer	415	82.7
Government employee	48	9.5
Merchant	38	7.6
Others	1	0.2
Head of the household		
Male	493	98.2
Female	9	1.8
Number of under 5 children in the household		
One	244	48.6
Two or more	258	51.4

**Table 2:** Household agricultural production and food security status in the Aleta Wondo district, Southern Ethiopia.

Variables (*n* = 502)	Frequency	Percentage
Agroecology		
Midland	402	80.1
Highland	100	19.9
Major source of food		
Domestic production	389	77.5
Market	113	22.5
Proportion of households who grew		
*Ensete* (*false banana*)	489	97.4
Cash crops	439	87.5
Vegetables	384	76.5
Cereals	302	60.2
Legumes	48	9.6
Own livestock		
Yes	340	67.7
No	164	32.3
Household food security status		
Secure	259	51.6
Mild insecurity	67	13.3
Moderate insecurity	134	26.7
Severe insecurity	42	8.4

**Table 3:** Outputs of the linear regression analysis on factors associated with the dietary diversity score among children aged 6–23 months, Aleta Wondo, Southern Ethiopia.

Variables and coding schemes	Bivariate		Multivariate	
	*β*	*p* value	*β*	*p* value
Proximate variables				
Household food security status (1–4)	−0.14	0.002^*∗*^	−0.13	0.001^*∗*^
Knowledge of mothers on IYCF (1–10)	0.19	0.007^*∗*^	0.21	0.004^*∗*^
Fathers involvement in IYCF (1–8)	0.34	0.028^*∗*^	0.32	0.016^*∗*^
Distal variables				
Age of mothers (15–49 years)	0.16	0.052	0.09	0.216
Wealth index (1–5)	0.06	0.018^*∗*^	0.54	0.041^*∗*^
Father's education (0 = illiterate and 1 = literate)	0.94	0.003^*∗*^	0.48	0.002^*∗*^
Mother's education (0 = illiterate and 1 = literate)	0.31	0.035^*∗*^	0.30	0.243
Ownership of backyard garden (0 = no and 1 = yes)	0.48	0.004^*∗*^	0.38	0.010^*∗*^
Mothers involvement in income generating (0 = no and 1 = yes)	0.26	0.045^*∗*^	0.13	0.162
Age of child in months (6–23)	0.15	0.001^*∗*^	0.04	0.001^*∗*^
Participation in food demonstration (0 = no and 1 = yes)	0.25	0.011^*∗*^	0.19	0.036^*∗*^
Received IYCF information via mass media (0 = no and 1 = yes)	0.22	0.007^*∗*^	0.16	0.015^*∗*^
Received IYCF information during ANC (0 = no and 1 = yes)	0.94	0.035^*∗*^	0.91	0.022^*∗*^
Received IYCF information during PNC (0 = no and 1 = yes)	0.27	0.009^*∗*^	0.21	0.043^*∗*^
Livestock ownership (0 = no and 1 = yes)	0.75	0.200	−0.95	0.320

^*∗*^Significant association at *p* value of 0.05.

## Data Availability

The dataset analyzed is available from the corresponding author on reasonable request.

## Abbreviations

ANC: Antenatal care
ASL: Above sea level
CI: Confidence interval
DALYs: Disability-adjusted life years
DD: Dietary diversity
DDS: Dietary diversity score
DHS: Demographic and health survey
FANTA: Food and nutrition technical assistance
HFIAS: Household food insecurity and access scale
HDA: Health development army
HEWs: Health extension workers
IRB: Institutional review board
IYCF: Infant and young child feeding
MOH: Ministry of Health
NNP: National Nutrition Program
PCA: Principal component analysis
PNC: Postnatal care
SD: Standard deviation
SPSS: Statistical package for social science
SSA: Sub-Saharan Africa
VIF: Variance inflation factor
WHO: World Health Organization.

## Supplementary Materials

Supplementary Materials Supporting file 1: questions used for assessing mother's knowledge of IYCF.
