# Supplementary material for: Dietary Diversity among Children Aged 6–23 Months in Aleta Wondo District, Southern Ethiopia
Source: J Nutr Metab. 2019 Nov 13;2019:2869424. doi: 10.1155/2019/2869424 (PMC6878804; doi:10.1155/2019/2869424)
Supplement: Supplementary Materials — Supporting file 1: questions used for assessing mother's knowledge of IYCF. [file 2869424.f1.docx]

**Ten questions used for assessing mothers’ knowledge of IYCF.**

|  | Question | Choices |
| --- | --- | --- |
| 1 | Do you know for how many months to breast feed your child? | 1.Yes  2. No |
| 2 | Do you know that when you start complementary feeding for your child? | 1.Yes  2. No |
| 3 | Do you know that for how long do you continue feeding complementary food for your child? | 1.Yes  2. No |
| 4 | Do you know what types of foods should be given to your child every day? | 1.Yes  2. No |
| 5 | If, yes list the type of foods that you know? | 1.Grains, roots and tubers  2. Legumes and nuts  3. Dairy products  4. Flesh foods  5. Eggs  6. Vitamin-A rich fruits and vegetables  7. Others fruits and vegetables |
| 6 | Do you think egg should be given to your child? | 1.Yes  2. No |
| 7 | Do you think meat should be given to your child? | 1.Yes  2. No |
| 8 | Do you think milk and milk products/excluding breast milk should be given to your child? | 1.Yes  2. No |
| 9 | Do you think breastfeeding should be continued after 1 year of age? | 1.Yes  2. No |
| 10 | How many times should children receive food in a typical day? | ________times |
